# Open-source controller for dynamic cardiovascular models

**DOI:** 10.1016/j.ohx.2023.e00500

**Published:** 2023-12-12

**Authors:** Muhammad Farooq, Muhammad Riaz ur Rehman, Patricia Vazquez, William Wijns, Atif Shahzad, Marcin J. Kraśny

**Affiliations:** a Smart Sensors Lab, The Lambe Institute for Translational Medicine, School of Medicine, College of Medicine, Nursing and Health Sciences, University of Galway, Ireland; b Translational Medical Device Lab, The Lambe Institute for Translational Medicine, School of Medicine, College of Medicine, Nursing and Health Sciences, University of Galway, Ireland; c Centre for Systems Modelling and Quantitative Biomedicine (SMQB), University of Birmingham, B15 2TT, United Kingdom

**Keywords:** Pressure controller, Pulse duplicator, Pulsatile pump, Blood pressure phantom, Fatigue test

## Abstract

Cardiovascular pressure sensors require dedicated, reliable, and customisable performance testing equipment. Devices available on the market, such as pulsatile pumps and pulse multipliers, offer limited adaptability to the needs of pressure sensor testing or are highly complex tools designed for other purposes. Therefore, there is a strong need to provide an adaptable and versatile device for characterisation during prototype development, prior to animal model testing. Early development requires detailed characterisation of a sensor performance in a realistic environmental scenario. To address this need, we adapted an off-the-shelf pressure chamber with a custom Arduino-based controller to achieve a rapid change in pressure that simulates the pulsatile profile of human blood pressure. The system is a highly customisable tool, and we have experimentally shown that it works successfully in a wide range of pressures from 30 mmHg to 400 mmHg with a resolution of 2 mmHg. By adjusting the chamber volume using a water balloon, we achieved a cycle rate of up to 120 beats per minute. The device can be operated directly from the Arduino IDE or with a customised graphical user interface developed by our research group. The proposed system is intended to assist other researchers in the development of industrial and biomedical pressure sensors.

## Graphical abstract

**Figure:**
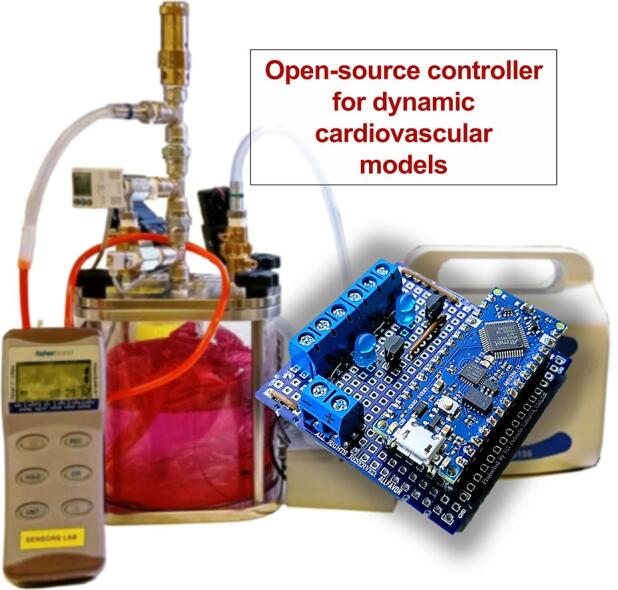

**Specifications table**.

**Table:** 

Hardware name	Pulsatile Pressure Chamber Controller
Subject area	• Engineering and materials science • Medical (e.g., pharmaceutical science) • Educational tools and open-source alternatives to the existing infrastructure
Hardware type	• Measuring physical properties and in-lab sensors • Field measurements and sensors • Mechanical engineering and materials science
Closest commercial analog	Pulsatile Blood Pump Pulse Duplicator, Pulse Multiplier
Open source license	Arduino source code and the accompanying material: **MIT** LabView GUI design files: **MIT** The data set and figures are licensed under **CC0-1.0**
Cost of hardware	∼1000 EUR dedicated pressure chamber with solenoid valves ∼350 EUR pressure pump ∼350 EUR required parts and components (Not including PC)
Source file repository	OSF project repository: https://doi.org/10.17605/OSF.IO/5D4PH

## 1 Hardware in context

Intracranial, intraocular, and cardiovascular pressures are essential physiological signals monitoring of which can aid in the early detection, treatment, and prevention of human diseases [1], [2], [3]. During the last few years, a great variety of wearable and implantable pressure sensors for monitoring different physiological pressures [2], [4], [5], [6], [7] have been reported.

All these sensing solutions require, during their development phase, detailed performance characterisation, first in the laboratory setting and later in animal and clinical trials.

The logical first step in the development of a cardiac pressure sensor is to characterise its response under cyclic pressures close to the human blood pressure profile. The human pulsatile blood pressure waveform is described using a few core parameters within a single cycle. Specifically, the waveform changes from a minimum value, which is known as Diastolic Pressure (DP), to a peak called Systolic Pressure (SP), and then decreases to Dicrotic Notch Pressure (DNP) to increase again to Dicrotic Peak Pressure (DPP), and finally returns to DP [8], [9]. The difference between SP and DP is known as Pulse Pressure (PP) [10]. The commonly accepted values for normal blood pressure expressed in millimetres of mercury (mmHg) are 120, 80, 90, and 100 for SP, DP, DNP, and DPP, respectively. In extreme cases, however, the systolic pressure can rise to 370 mmHg, and the diastolic pressure can drop to 30 mmHg [10]. Another important factor to consider when designing a pressure sensor is long-term durability under difficult conditions of cyclic loading. The typical blood pressure cycle for a healthy adult is approximately 60–100 times per minute [11]. This means 2.6–4.3 million beats within one month only. These parameters make designing a blood pressure sensor a challenging task that requires an extremely rigorous lab testing phase.

Due to these rapid changes within a single blood pressure cycle, the key requirements for pressure sensors are (i) the response time required to detect changes, (ii) stability over time, and (iii) durability. Therefore, before testing in animal models, these devices can be studied in a i.e., pressure chamber mimicking normal and/or extreme pulse pressure conditions. The durability and long-term performance of the sensors can be tested by fatigue testing [6], which requires long-lasting exposure of the sensor to oscillating pressure conditions.

Universal testing device rigs such as available pulsatile pumps could be applied, however the most common models offer limited configurability options to allow sensor testing in extreme case parameters, i.e., pressure control is of limited adjustability, and achieving a 1:1 pulse profile replica is often not possible. More advanced tools, such as pulse duplicators or pulse multipliers, are designed to simulate cardiac physiological stress, and are most commonly used for testing prosthetic valve fatigue [12]. Several commercial and scientific pulse duplicators are available on the market. For example, Rajeev *et. al.* [12] reported a custom-made pulse duplicator for a simulated cardiac environment for valve testing, but this system is only adjustable with the exchange of hardware components. Software-based cardiac simulator systems designed in LabVIEW were reported by Cole *et al.* [13] and Bazan *et al.* [14]. Available pulse duplicators are complex and costly devices dedicated to the development of advanced cardiac implants and are impractical for the purpose of testing pressure sensors.

In this work, we propose an open-source controlled pressure chamber system for testing biomedical pressure sensors. The controller is a simple yet reliable Arduino-based system, developed as a versatile tool, which allows a high level of customisation and adaptability to the user’s specific needs. We present instructions to alter the commercial pressure chamber with electrically controlled solenoid valves, to use the system as a dedicated rig for the development of pressure sensors, their characterisation, and fatigue testing. Finally, for ease of operation of the system, we propose a LabVIEW windows-based Graphical User Interface (GUI) to control device operation.

## 2 Hardware description

**Fig. 1:**
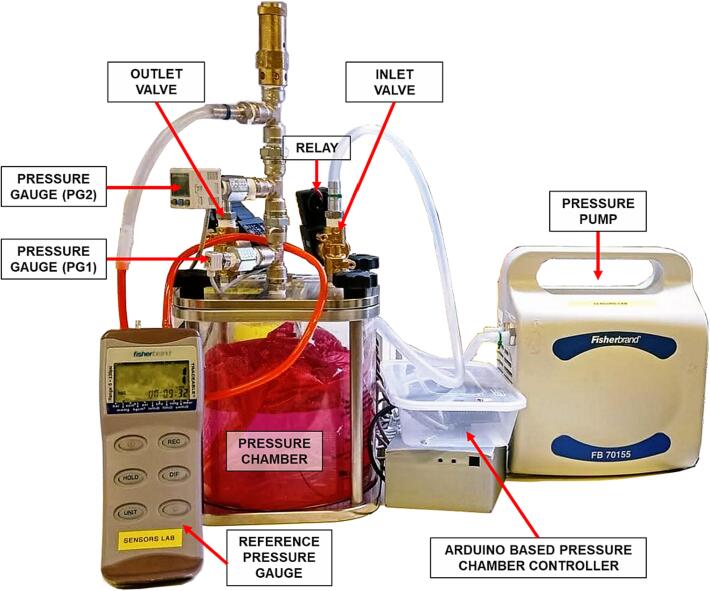
General overview of the components of the pressure chamber system proposed in this manuscript.

The proposed system comprises a pressure chamber (SR-TEK CT series) connected to a pressure pump (Fisherbrand FB 70155). The pressure inside the chamber is controlled by two valves (L121V02), an inlet and an outlet, operated by ZA10AF1 relays (see Fig. 1). For the measurement of pressure inside the chamber, we use a pressure gauge SMC PSE543-N01 (PG1), with its voltage output connected to the analogue input of an Arduino Nano Every board. Additionally, visual pressure readings are provided by an optional pressure gauge, SMC ZSE30AF-01-N (PG2), used as reference for pressure values. This meter can also be used for the purpose of controller calibration, as explained in the *Validation and characterization* section. The system’s logic is implemented in the custom firmware and hardware integrated in the Arduino platform.

**Fig. 2:**
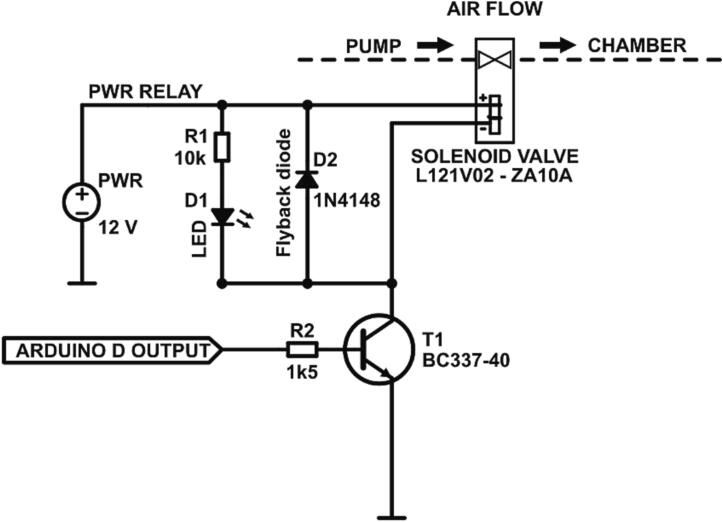
Relay switching circuit topology.

Such a system allows the regulation of pressure in the chamber in the range of −30 to 100 kPa (-220 to 775 mmHg) with the voltage output of the pressure gauge (PG1) in the range 1 to 5 V. To increase the stability of the pressure measurement, we use Arduino’s internal voltage reference of 4.3 V, which restricts the upper limit of the pressure to 65 kPa (487.5 mmHg). Within this range, the 10-bit resolution provides 1024 discrete steps. Therefore, each step corresponds to 4.2 mV (4.3 V/1024), which translates to a resolution of 1.58 mmHg throughout the entire measurement range. Relays are controlled with a transistor circuit operated from the digital output pins of the Arduino board, as shown in the circuit schematic Fig. 2.

At 12 V power supply, ZA10AF1 requires ∼200 mA of current in the ON stage, which is within the limits (800 mA) of the BC337 transistors (T1) used in the control circuit. Moreover, the pulsatile operation of the device allows for additional transistor cooling time for reliable operation. To dissipate energy in the relay coils for the switching-off phase, a standard 1N4148 signal diode is used as a flyback diode (D2). For visual indication of the working valve (inlet or outlet), an additional blue LED (D1) with the appropriate current limiting resistor (R1) is added in parallel to the solenoid coil circuit. The full circuit schematic is available in the project repository, see *Design files summary* section for details.

To control the chamber from the PC, we implemented a simple version of communication commands inspired by the Standard Commands for Programmable Instruments (SCPI). This allows the user to access and modify all chamber parameters during operation. The resulting system can be controlled directly from the Arduino IDE using the Serial Monitor to access and change controls on the fly, without interfering with the system operation. Additionally, for more convenient use, we have developed a dedicated Graphical User Interface (GUI) software as a standalone application in LabVIEW 2012. Finally, all Arduino readings of the sensed pressure are sampled at a fixed (user-adjustable) sampling rate, allowing for precise signal reconstruction and time calculation of the chamber operation. This is described in more detail in later sections.

The core software and hardware solutions implemented in this manuscript allow others to use and/or apply our work in similar projects with requirements listed below:

• simple, yet reliable, and highly customisable hardware circuit for relay control.
• software control with communication commands (SCPI inspired communication for Arduino).
• reliable sensor readings with reference voltage.
• precise signal sampling rate to allow time control of the signal.
• device fatigue testing.

**Design files summary**.

**Table:** 

Design file name	File type	Open source license	Location of the file
Pressure-chamber-circuit-schematic.pdf	Circuit schematic	MIT	https://doi.org/10.17605/OSF.IO/5D4PH
SSL_PressureChamber_FW	Arduino Firmware	MIT	https://doi.org/10.17605/OSF.IO/5D4PH
SSL_PressureChamber_GUI	LabView	MIT	https://doi.org/10.17605/OSF.IO/5D4PH
Figures (all figures used in the paper)	Figure (PNG/PDF/SVG)	CC BY-SA	https://doi.org/10.17605/OSF.IO/5D4PH
Data	Dataset (Excel file)	CC BY-SA	https://doi.org/10.17605/OSF.IO/5D4PH

**Bill of materials summary**.

The table below presents all circuit components used in our system with suppliers’ price lists in euro including 23 % VAT. The reported prices are for 2022.

**Table:** 

Designator	Component	Number	Cost per unit - currency [EUR]	Total cost - Currency [EUR]	Source of materials	Material type
Dedicated pressure chamber with solenoid valves	SR-TEK CT Series, 3 Litres	1	1000.00	1000.00	Smartreservoirs, UK	Other
Pressure Gauge (PG1)	SMC, PSE543-N01	1	90.00	90.00	Radionics Ltd	Other
Pressure Gauge (PG2)	SMC, ZSE30AF-01-N	1	110.00	110.00	Radionics Ltd	Other
Pressure Pump	Fisherbrand FB 70155	1	350.00	350.00	Fisher scientific	Other
Arduino	Arduino Nano Every	1	18.00	18.00	Radionics Ltd	Other
Transistor (T1)	BC337-40	2	0.14	0.28	Radionics Ltd	Semiconductor
LED (D1)	C566DBFECU0W0351	2	0.23	0.46	Mouser	Semiconductor
Flyback diode (D2)	1N4148	2	0.12	0.24	Mouser	Semiconductor
Resistor (R1)	10 kΩ, SMD 0805, 1 %	2	0.12	0.24	Mouser	Other
Resistor (R2)	1.5 kΩ, SMD 0805, 1 %	2	0.12	0.24	Mouser	Other
12 V Power Supply	RS Stock No.: 175–3303	1	10.00	10.00	Radionics Ltd	Other
Prototype breadboard	RS Stock No.: 897–1638	1	2.50	2.50	Radionics Ltd	Other
Terminal block 2 PIN	PCB straight	1	1.00	1.00	Mouser	Other
Terminal block 3 PIN	PCB straight	3	1.10	3.30	Mouser	Other
Hose Nipple (HN)	RS Stock No.: 499–3732	2	5.50	11.00	Radionics Ltd	Other
Tee Threaded Adapter (TA)	RS Stock No.: 367–5922	3	14.50	43.50	Radionics Ltd	Other
Straight Threaded Adapter (SA)	RS Stock No.: 807–564	3	6.20	18.60	Radionics Ltd	Other
Threaded Reducer (TR)	RS Stock No.: 807–413	1	10.00	10.00	Radionics Ltd	Other
Sealing tape	PTFE Tape RS Stock No.: 231–964	1	2.30	2.30	Radionics Ltd	Other
Flexible Pipes	Flexible tube; 1 m, 10 mm outer diameter	1	10.00	10.00	Fisher scientific	Other

## 3 Build instructions

In this section, the reader will find instructions on how to assemble the device. All required components are listed in the *Bill of materials summary* (BOM) section and the required software tools are listed in the *Design files summary* section.

### 3.1 Hardware

**Fig. 3:**
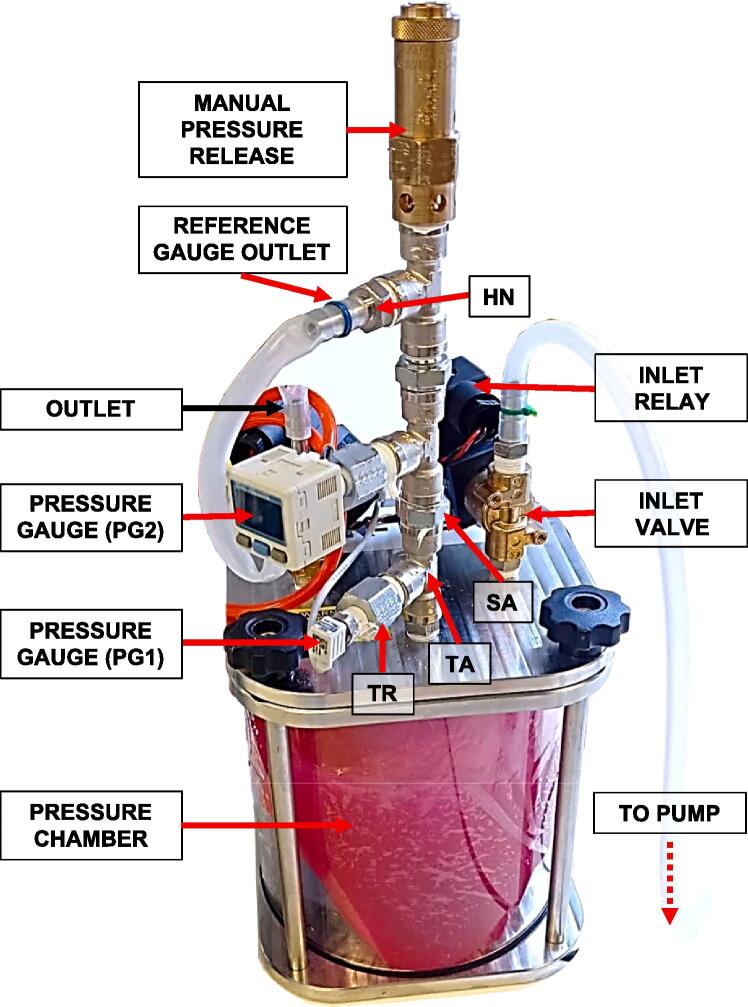
Pressure chamber with piping, connectors, solenoid valves, and pressure gauges.

Connect the pressure pipes to the chamber according to the diagram shown in Fig. 3. Both pressure gauges (PG1 & PG2) are connected via Threaded Reducers (TR) to the main piping system assembled from two Tee Threaded Adapters (TA) and two Straight Threaded Adapters (SA). As shown in Fig. 3., we added one extra (optional) set of TA & SA to connect a third pressure outlet, which we used for calibration as described in the *Validation and characterisation* section. On top of the pipe there is a screw valve (delivered with the pressure chamber), which can be operated manually and used, e.g., emergency pressure release if necessary. All metallic pipe connections are sealed with PTFE sealing tape.

The pump outlet should be connected to one of the solenoid valves, while the outlet valve (exhaust valve) can remain open. If the exhaust valve releases pressure too quickly for your application, consider partially blocking the exhaust.

An alternative to the pressure chamber presented in this paper could be the use of other sealed container (i.e., bottle) with a plastic cup adjusted for required pipe connection. Such chamber system, without active pressure control, was used in our previous work [5]. Solenoid valves with required adapters could be then mounted alongside the pipelines.

**Fig. 4:**
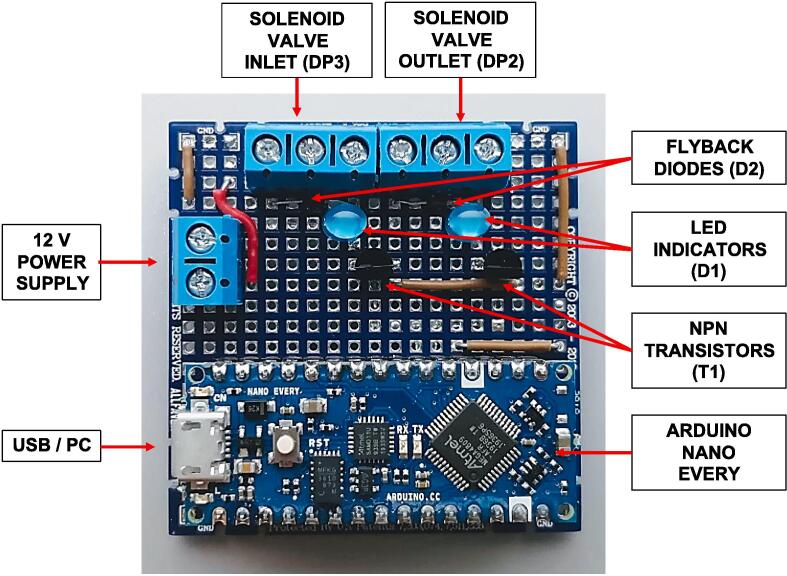
Assembled controller, overall dimensions: 5x5 cm, height 3.5 cm. The terminal block for the pressure gauge input is placed at the bottom, therefore it is not visible in this view. A detailed wiring diagram is available as a circuit schematic in the supplementary data.

The controller board should be soldered according to the circuit diagram shown in Fig. 2 and the full circuit schematic is available in the project repository. From the Arduino digital pins (DP) we use DP2 as the outlet valve and DP3 as the inlet valve. The Arduino is connected to a PC with a standard micro-USB cable, and 12 V power supply should be connected to the board via one of the terminal blocks. Both pressure gauges are connected to a 12 V power supply, so only one power supply is required for the device operation. While soldering and assembling the circuit, extra care should be taken to keep 12 V power lines insulated and away from the Arduino connections. In addition, for safe operation of the device, all 12 V power connections (i.e., on solenoid valves) should be insulated, and ideally, the controller should be encapsulated in the enclosure. The assembled controller is shown in Fig. 4.

## 4 Software

Install Arduino IDE, connect your Arduino controller board with the right options from the Tools menu (Board and Port), compile and upload the sketch (SSL_PressureChamber_FW) to the Arduino.

Test functionality by connecting with the COM port communicator (Tools/Serial Monitor) and type:

**Table:** 

IDF

Arduino controller will return the version of the firmware:

**Table:** 

Smart Sensors Lab Pressure Chamber Controller ver.1.00.08

as shown in Fig. 5.

**Fig. 5:**
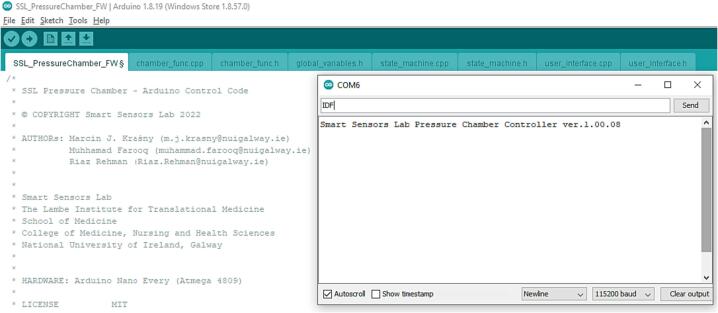
Arduino IDE Serial Monitor, example of communication with the Smart Sensors Lab Pressure Chamber Controller.

The pressure chamber can now be operated manually from the Arduino IDE using text commands typed in the Arduino Serial Monitor, i.e.:

**Table:** 

DIASTOLIC, 42

changes diastolic pressure to 42 mmHg.

A list of core parameters with their short descriptions is presented in Table 1. A list of all parameters accessible to the user is available in the Arduino firmware repository.

**Table 1:** List of core commands used to control the Arduino controller with their short description.

Parameter command	Value	Unit	Comment
IDF	–	–	Identification query, returns the firmware version
PARAMS	–	–	Returns the current values of all parameters (including internal params)
BP PARAMS	–	ADC, mmHg	Returns current settings of pressure parameters in ADC and mmHg units
ADC INTERVAL	xxx	µs	Time in microseconds [µs] between consecutive pressure measurements
PRESSURE	–	ADC, mmHg	Returns current chamber pressure
DIASTOLIC	xxx	mmHg	Set the diastolic pressure value to xxx
SYSTOLIC	xxx	mmHg	Set the systolic pressure value to xxx
DICROTIC NOTCH	xxx	mmHg	Set the dicrotic notch value to xxx
DICROTIC PEAK	xxx	mmHg	Set the dicrotic peak pressure value to xxx
START	–	–	Start pressure cycles
STOP	–	–	Stop pressure cycles

The user can perform fatigue testing by running the device in a cyclic manner. Two counters will be updated, and the cycle count number will be stored in the non-volatile memory (EEPROM) of the microprocessor. One counter value is for the overall cycles of the chamber (this can be of help to investigate troubleshooting and fatigue of the relays/chamber itself), while the second one can be zeroed by the user and kept increasing throughout the cycle of the experiment.

### 4.1 GUI

For easier device operation, we developed dedicated GUI software in LabVIEW 2012. The GUI is available as standalone Windows software, and the user can follow simple installation instructions from the self-extracting archive. The operation of the GUI is explained in the *Operation instructions* section.

### 4.2 Operation instructions

As described in the previous sections the user can operate the controller directly from the Arduino IDE or can use our custom design GUI developed in LabView.

### 4.3 General operation algorithm and logic (state machine) of the Arduino controller

**Fig. 6:**
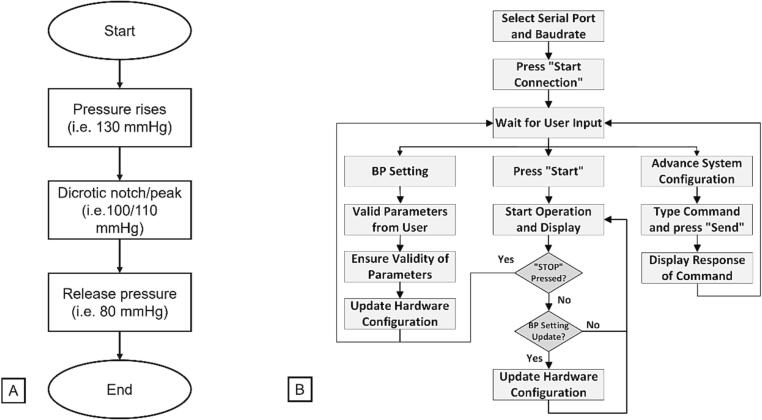
Arduino flow diagram of firmware (a) GUI flow diagram of operation control (b).

The general operation of the Arduino firmware follows the linear logic of a state machine implemented within the switch case loop, as shown in the flow diagram in Fig. 6a. After receiving the “START” command, Arduino measures chamber pressure at cyclic intervals and controls the operation of the inlet & outlet valves to adjust chamber pressure to the desired values set by the user. To increase time and save some operational Arduino power, the state machine loop operates by comparing raw ADC values read from the pressure gauge with the Arduino’s ADC. Translation from pressure values in mmHg to ADC values occurs only once when initial parameters are given by the user (or if they are changed during the device operation). Analogously, the user is given chamber pressure values in real time (displayed in GUI), but translation of ADC values to pressure expressed in mmHg occurs on the PC side, as explained in the next section. Chamber runs until the Arduino controller receives “STOP” command from the user. For safety reasons, a simple error handling mechanism is implemented in the Arduino firmware. This operates on a time intervals basis, and if no state change occurred for a set time (user defined), the controller goes into emergency mode. All relays are then opened, and pressure from the chamber is released. The user is notified of the error mode by fast flashing of the Arduino’s built-in diode.

### 4.4 GUI software operation

**Fig. 7:**
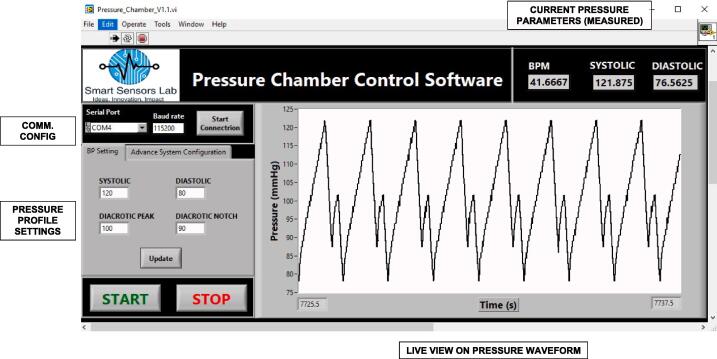
Front panel of the GUI application window overview with marked serial port definition and communication settings with details on chamber cycle parameters with tab for Advanced system configuration allowing easy access to all the controller features with the use of communication protocol.

We have developed a windows based application for the user interface to control the pressure chamber. The general flow of the device operation is shown in Fig. 6b., and the application interface is shown in Fig. 7.

First, the user specifies the valid serial port and baud rate for serial communication with the hardware as shown in Fig. 6b. The user then provides custom values for the desired pressure waveform on the Pressure Profile Settings tab. The valid combination must meet the following conditions: systolic > dicrotic peak > dicrotic notch > diastolic. The update button sends new values to the Arduino controller. In the case of an invalid combination, an error message is displayed, and the user can provide the valid combination and click the refresh button again. The user can select the Advanced System Configuration Settings tab to configure the hardware via text commands. The user can enter a hardware command in the command input text box. Pressing the send button sends the command to the hardware, and the device’s response is displayed in the Serial Read field.

The current pressure parameters are displayed in the right upper corner of the program interface. This includes calculated beats per minute (BPM), systolic and diastolic pressure values from the pressure waveform data streamed directly from the Arduino controller. The pressure values are calculated from ADC codes received from the hardware with the use of the linear fit function described in the *Calibration* section. In the live view on pressure waveform, a pressure plot is shown to the user. By default, a 12 s window view is shown for an optimised overview of the current waveform. The software calculates the pressure parameters from the pressure measurements delivered by the pressure gauge. For valid calculation of current pressure parameters, a minimum of four full cycles are required.

## 5 Validation and characterisation

### 5.1 Calibration

**Fig. 8:**
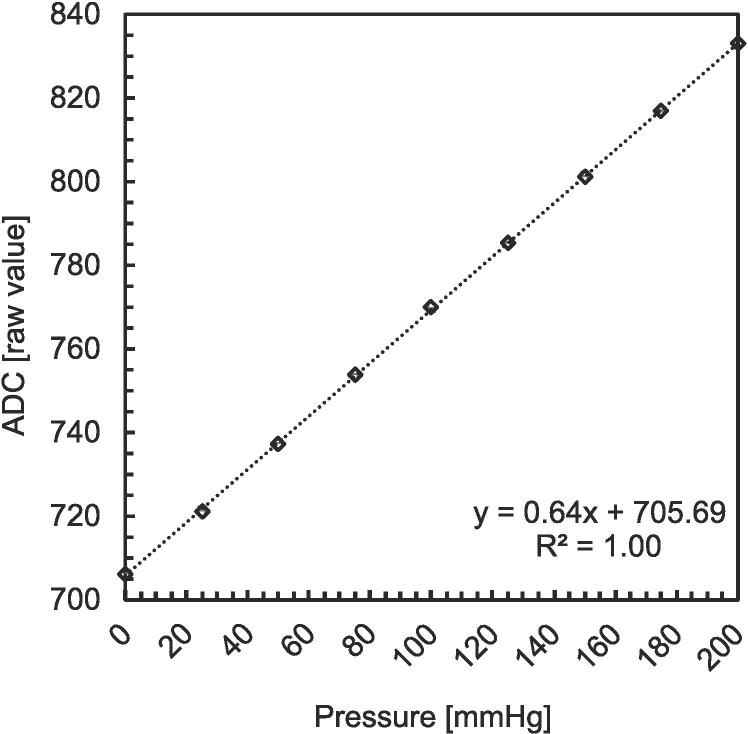
Calibration of PSE543-N01 pressure gauge.

To calibrate the PSE543-N01 (PG2) pressure gauge, the pressure inside the chamber was increased from 0 (atmospheric pressure) to 200 mmHg with 25 mmHg intervals. The pressure gauge signal was recorded as raw ADC values (0–1023 range) with the serial monitor of the Arduino IDE. The corresponding pressure values were recorded with the Fisher Traceable 06–664-21 handheld pressure gauge (shown in Fig. 1) connected to the reference pressure gauge outlet shown in Fig. 3. Alternatively, calibration procedure can be also done with the pressure gauge PSE543-N01 (PG1). As stated before, to avoid dependencies on Arduino power supply voltage, we use an internal reference voltage of 4.3 V. Fig. 8 shows the raw ADC values against the corresponding pressure values. As expected, the pressure gauge shows a linear relationship between the raw ADC values and the pressure in the chamber. We used a linear fit function (*y = ax + b*) to find the parameters of the linear relationship, where the slope was found to be 0.64, with a 95 % confidence interval (CI) of [0.633, 0.641] and an intercept at 705.69, 95 % CI [705.19, 706.20]. Later, we use this equation to exchange raw ADC values for the corresponding pressure values expressed in mmHg units. Calibration of repeatability with its statistical analysis is available to the reader in the supplementary dataset file (see section *Design files summary*).

### 5.2 Pressure profile with empty chamber vs variable amplitude of pulse pressure

The default values of SP, DP, DNP, and DPP are set to 120, 80, 90, and 100, respectively. When the “START” button is pressed, the outlet valve is closed, and the inlet valve allows the pressurisation of chamber to the set value of the SP. Then the state machine implemented in Arduino controls valves operation to keep the desired pressure profile as requested by the user. Fig. 7 shows an example of the default pressure profile. The chamber pressure increases from DP = 80 to SP = 120 and then decreases to DBP = 90, following by an increase to DPP = 100 and then again falling to the set value of DBP. These default values of SP and DP are common physiological arterial pressure values measured in the human body. The maximum value of SP, minimum value of DP and beats per minute (BPM) are shown in the upper right corner of the GUI. The chamber used in our study was adjusted to simulate a volume of one human lung (approximately 3 L). The pressure pump used in this setup has a capacity of up to 9.2 L/minute, thus pressure profile achieved with an empty chamber is limited to 42 BPM as shown in Fig. 7.

**Fig. 9:**
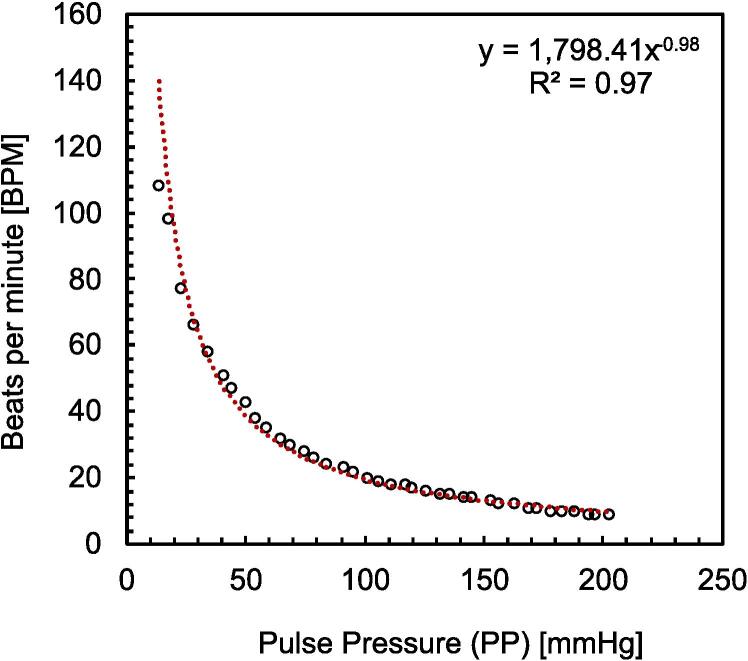
The relationship between the measured PP and BPM.

If one would require faster BPM values and keep the volume of the full chamber, a possible approach would be to decrease the Pulse Pressure (PP) amplitude. In the above example, PP is set to 40 mmHg (*120*–*80 = 40*). However, if the PP would be set to 10 mmHg only, the resulted BPM could be increased up to 100 BPM. The relationship between pulse pressure and beats per minute is shown in Fig. 9. The values of the SP were varied between 30 and 400 mmHg with an interval of 10 mmHg, and similarly DP was varied between 20 and 205 mmHg at an interval of 5 mmHg. All the values of both set and measured pressure parameters are available in the project Data file (see *Design files summary*). From Fig. 9, it can be noticed that there is an inversely proportional power relationship between the BPM and PP, which means that the smaller the PP the faster the cycle expressed in BPMs.

Please note that, the main factor influencing the speed of valves operation is their opening time, which according to datasheet is 20 ms [15]. Additionally, due to the use of the 10-bit internal ADC from the Arduino with 4.3 V reference source, we are limited to a pressure reading of 1 ADC corresponding to 1.58 (further approximated to 2) mmHg of sensing resolution.

### 5.3 Chamber volume vs beats per minute

An alternative method to increase the cycle speed (BPM) of the pulse profile is to reduce the chamber volume.

**Fig. 10:**
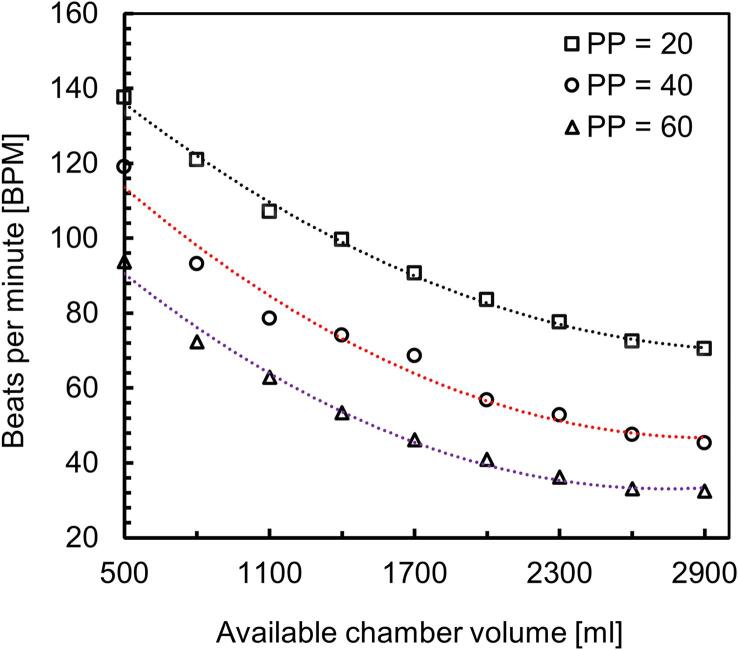
BPM at different available chamber volumes when PP = 20 (square markers), PP = 40 (circle markers), and PP = 60 (triangle markers). For all PP settings, dotted line, shows 2nd order polynomial fit.

To test this option, we conducted an experiment, where the volume of the pressure chamber was changed from empty (2900 ml) to 500 ml with an interval of 300 ml. To decrease the volume of free air in the chamber, we used balloons filled with water. An example with a balloon and two different water content in the chamber is shown in Fig. 1 and Fig. 3. This experiment was performed with three settings of PP (20 [SP = 100, DP = 80], 40 [SP = 120, DP = 80], 60 [SP = 160, DP = 80]). All the values of the measured BPM for each setting of PP are listed in the project Data file (*Design files summary*). Fig. 10 shows that there is a non-linear, inversely proportional relationship between PP and BPM. Second-order polynomial equations were well fitted with very high R^2^ values, as shown in Fig. 10. Of note, decreasing the chamber volume to only 500 ml, with the default pressure parameters as defined in section *Pressure profile with empty chamber* vs *variable amplitude of pulse* pressure allows achieving BPM values in the range of 120.

### 5.4 Summary and future work

This work has presented a pressure chamber system with a pulse profile and cycle speed that can be adjusted in multiple ways, as shown in the *Validation and characterisation* section, which includes the modification of pressure parameters and the available chamber volume. Two main limitations of the proposed solution are a result of the operational speed of the mechanical valves used, limited to 20 ms, and the relatively low speed of the pump used in the described system configuration. Moreover, the accuracy is limited by the resolution of Arduino’s ADC, allowing for measuring pressure chamber with +/− 2 mmHg.

While we believe that 2 mmHg resolution provides sufficient accuracy for the intended chamber application, those aiming for even greater precision can explore an alternative path. The initial step toward improvement entails selecting a more appropriate pressure gauge, like the PSE541, which is specifically designed for operating within a positive pressure range. This change would effectively double the sensitivity, resulting in a resolution of less than 1 mmHg, all without the need for any further modifications to the currently proposed setup. If additional enhancements are necessary, the next step would involve a dedicated signal amplification, combined with a conditioning circuit to adjust for the overall input level of the ADC. This approach can also be complemented by, for instance, using Solid Stay Relays to drive valves with an increased switching speed.

As future work we plan to develop a dedicated Printed Circuit Board (PCB) for the Arduino controller, with an additional feature to control the proportional solenoid valves for inlet and outlet air flow speed directly from the software. In addition, the next version of the system will include a safety feature to control the pressure pump operation. To complement this the software & firmware will include an error-handling procedure to improve the safe operation of the chamber.

### CRediT authorship contribution statement

**Muhammad Farooq:** Writing – review & editing, Data curation, Writing – original draft, Visualization, Investigation, Formal analysis, Validation, Software. **Muhammad Riaz ur Rehman:** Writing – review & editing, Visualization, Investigation, Formal analysis, Validation, Software. **Patricia Vazquez:** Writing – review & editing. **William Wijns:** Writing – review & editing. **Atif Shahzad:** Writing – review & editing. **Marcin J. Kraśny:** Data curation, Writing – original draft, Visualization, Investigation, Formal analysis, Validation, Conceptualization, Methodology, Software, Writing – review & editing, Hardware.

## Declaration of competing interest

The authors declare that they have no known competing financial interests or personal relationships that could have appeared to influence the work reported in this paper.
